# Associations between Rotating Night Shifts, Sleep Duration, and Telomere Length in Women

**DOI:** 10.1371/journal.pone.0023462

**Published:** 2011-08-10

**Authors:** Geyu Liang, Eva Schernhammer, Lu Qi, Xiang Gao, Immaculata De Vivo, Jiali Han

**Affiliations:** 1 Division of Gastroenterology, Beth Israel Deaconess Medical Center, Harvard Medical School, Boston, Massachusetts, United States of America; 2 Key Laboratory of Environmental Medicine Engineering of Ministry of Education, School of Public Health, Southeast University, Nanjing, China; 3 Channing Laboratory, Department of Medicine, Brigham and Women's Hospital and Harvard Medical School, Boston, Massachusetts, United States of America; 4 Department of Epidemiology, Harvard School of Public Health, Boston, Massachusetts, United States of America; 5 Ludwig Boltzmann Institute for Applied Cancer Research (LBI-ACR) and Applied Cancer Research-Institute for Translational Research (ACR-ITR) VIEnna/CEADDP, Vienna, Austria; 6 Department of Nutrition, Harvard School of Public Health, Boston, Massachusetts, United States of America; 7 Department of Dermatology, Brigham and Women's Hospital, Harvard Medical School, Boston, Massachusetts, United States of America; University of Valencia, Spain

## Abstract

**Background:**

Telomere length has been proposed as a marker of aging. However, our knowledge of lifestyle risk factors determining telomere length is limited.

**Methods:**

We evaluated the associations between years of rotating night shifts, self-reported sleep duration, and telomere length in 4,117 female participants from the Nurses' Health Study. Telomere length in peripheral blood leukocytes was determined by Real-Time PCR assay. Information on rotating night shifts and sleep duration was collected via questionnaires prior to blood collection. We used multivariable linear regression to investigate the associations between rotating night shifts, sleep duration, and telomere length.

**Results:**

Compared with women in the category (9 hours), those in the lowest category of sleep duration (≤6 hours) had a 0.12 unit decrease in z score after adjustment for age, BMI and cigarette smoking (equivalent to 9-year telomere attrition, P for trend  = 0.05). Significant positive association between sleep duration and telomere length was seen among women under age of 50 (P for trend  = 0.004), but not among those over 50 (P for trend  = 0.33) (P for interaction  = 0.005). In addition, we observed that women with a longer history of rotating night shifts tended to have shorter telomere length, but this relation was not statistically significant (P for trend  = 0.36).

**Conclusion:**

We found that sleep duration was positively associated with telomere length among women under 50 years old. Further research is needed to confirm the observed associations.

## Introduction

Telomere length has been reported to have important influence on various chronic diseases and aging [1], and previous data have suggested it as a potential biomarker of risk of cardiovascular disease and several cancers [2]-[5]. However, little is known about lifestyle factors that determine telomere length. A recent study showed that the current and long-term full-time work was associated with shorter telomere length in women of the Sister Study [6]. Another report found that telomere length was shorter in patients with sleep apnea syndrome than in controls [7]. This suggests that work schedule and sleep situation may have an effect on telomere length. Rotating night shifts work and sleep deficits disrupt circadian rhythms and have important effects on health. Increasing evidence suggests that rotating night shifts and short sleep duration are potential risk factors for various metabolic disorders, cardiovascular disease, and cancers [8]–[12]. It is interesting that a history of extended periods of rotating night shifts work and short telomere length in peripheral blood leukocytes (PBL) were both associated with a decreased risk of melanoma in our previous studies [13], [14] and with lower risk of Parkinson's disease by us and other researchers [15], [16]. Short sleep duration was also associated with a lower risk of Parkinson's disease [15]. To our knowledge, there are no epidemiologic data on the potential associations between rotating night shifts, sleep duration, and telomere length in a large general population. Here we evaluated these associations in a large ongoing US cohort of women: the Nurses' Health Study (NHS).

## Methods

### Ethics Statement

This study was approved by the Human Research Committee at the Brigham and Women's Hospital (Boston, MA) with written informed consent from all participants.

### Study population

The NHS was established in 1976 among 121,700 female registered nurses between the ages of 30 and 55. Participants completed biennially mailed questionnaires to update information on exposure status and newly diagnosed diseases. Between 1989 and 1990, blood samples were collected from 32,826 cohort members and were stored with liquid nitrogen. Between 2008 and 2010, several nested case-control studies were conducted within the NHS blood cohort to investigate the association between telomere length and stroke [17], myocardial infarction [18], breast cancer [19], skin cancer [14], and endometrial cancer [20]. We used all the controls from these nested case-control studies that had previously been analyzed for telomere length. The samples were limited to Caucasian women. A total of 4,117 women formed the baseline population for this study.

### Assessment of sleep duration, rotating night shifts and covariates

Sleep duration was asked in the 1986 questionnaire, corresponding to the total hours of actual sleep in a 24-hour period. Seven options were provided (hours): 5 or less, 6, 7, 8, 9, 10, 11 or more. Information on rotating night shifts was collected from the 1988 questionnaire. Participants were asked for their total number of years of rotating night shifts, which was characterized as “at least three nights per month in addition to working days or evenings in that month”. Eight categories were provided (in years): never, 1–2, 3–5, 6–9, 10–14, 15–19, 20–29, 30 or more. Information on age, cigarette smoking (pack years) and body mass index (BMI) at blood draw was also collected.

### Telomere length measurement

Genomic DNA was extracted from peripheral leukocytes using the QIAmp 96-spin blood protocol. The relative average telomere length was determined by a high-throughput 384-well Real-Time PCR assay using the Applied Biosystems 7900HT PCR System. The T/S ratio (-dCt) for each sample was calculated by subtracting the average 36B4 Ct value from the average telomere Ct value. The relative T/S ratio (-ddCt) was determined by subtracting the T/S ratio value of the 5 ng standard curve point from the T/S ratio of each unknown sample. Blinded quality-control samples were interspersed throughout the sets to assess inter-plate and intra-plate variability. The coefficients of variation (CV) of the telomere Ct and 36B4 Ct were lower than 5% in all nested case-control studies.

### Statistical analysis

We calculated a z score of telomere length among controls of each nested case-control study and pooled the z scores for further analysis. The z scores were categorized into quintiles. Multivariable linear regression models were used to examine the relationships between rotating night shifts or sleep duration and telomere length. Age, BMI, and cigarette smoking (pack years) were adjusted in the models. P trends across categories were calculated in the regression models.

Because prolonged sleep (10+ hours) may reflect some adverse healthy conditions, we kept it as a single group and did not include it in the trend test. To test potential interaction between these sleep variables and age on telomere length, we modeled age as a dichotomous variable (50 years old as a cutoff point) and rotating night shifts or sleep duration as a continuous variable. The age of 50 years was used as a cutoff to separate pre and post-menopausal women [21]. We then tested a single multiplicative interaction term by the likelihood ratio test, comparing the model with the single interaction term with the model containing just the main effects of rotating night shifts or sleep duration and age variables, along with the same covariates. Statistical analyses were conducted using SAS software (version 9, SAS Institute, Cary, NC). All statistical tests were two-sided.

## Results

The characteristics of women by quintiles of telomere length are presented in Table 1. A significantly inverse relationship was found between the telomere length and age (P<0.0001), which is consistent with published data [2]. Women with shorter telomere length had a slightly higher BMI; this was significant after adjusting for age (P = 0.03). Women with shorter telomere length were more likely to smoke, but this was not statistically significant (P = 0.07). There was no significant association between menopausal status and telomere length.

**Table 1:** Characteristics of women by quintiles of telomere length.

	Quintile of telemere length (z score)				
	1	2	3	4	5
Age at blood draw (years)^a^	59.9	59.6	59.0	58.6	58.5
BMI at blood draw (kg/m^2^)^b^	25.6	25.4	25.4	24.9	25.2
Current smoking at blood draw (%)^c^	15.9	20.1	15.0	16.1	14.2
Menopausal statue (%)^d^					
Premenopausal	11.7	12.0	12.0	15.1	15.8
Postmenopausal	88.3	88.0	88.0	84.9	84.2
Rotating night shifts (years)	3.8	3.7	3.5	3.4	3.4
Sleep duration, ≤6 hours (%)	29.0	26.3	26.6	25.8	25.2

a P<0.0001.

b P = 0.03 after adjusting for age.

c P = 0.07 after adjusting for age.

d P = 0.81 after adjusting for age.

The relationships between telomere length, nightshift work, and sleep duration are presented in Table 2. After multivariate adjustment for age, body mass index, and cigarette smoking, women with 9 hours of sleep had Z score of 0.07, whereas those with less than 6 hours of sleep had telomere length Z score of -0.05 (equivalent to 9-year telomere attrition, P for trend  = 0.05). Women in the top category (≥10 h) had shorter telomere length (Z score, -0.10). We observed a significant interaction between sleep duration and age on telomere length (P for interaction  = 0.005) (Table 3). In the stratified analyses by age, the significant association between sleep duration and telomere length was found only among women under 50 years old (P for trend  = 0.004). The top category of sleep duration (9 h) was associated with a 0.75 unit increase in z score compared with the lowest category of sleep duration (≤6 h). In contrast, no significant association was found between sleep duration and telomere length among women more than 50 years old (P for trend  = 0.33).

**Table 2:** The relationship between telomere length (z score), rotating night shifts, and sleep duration.

	n	Mean±SE^a^ (z score)	P for trend a	Mean±SE^b^ (z score)	P for trend b
Rotating night shifts(years)					
Never	1583	−0.007±0.03	0.28	−0.008±0.03	0.36
1–2	965	0.04±0.03		0.04±0.03	
3–5	669	0.004±0.04		−0.003±0.04	
6–9	304	0.03±0.06		0.03±0.06	
10–19	305	−0.07±0.06		−0.06±0.06	
≥20	166	−0.05±0.08		−0.05±0.08	
Sleep duration (hours)					
≤6	1026	−0.04±0.03	0.06^c^	−0.05±0.03	0.05^c^
7	1703	0.007±0.02		0.01±0.02	
8	948	0.03±0.03		0.02±0.03	
9	167	0.07±0.08		0.07±0.08	
≥10	19	−0.04±0.23		−0.1±0.23	

a Adjusted for age (continuous variable).

b Adjusted for age (continuous variable), BMI (continuous variable), and cigarette smoking (pack years).

c P for trend from sleep duration ≤6 hours to 9 hours.

**Table 3:** The relationship between telomere length (z score), rotating night shifts, and sleep duration stratified by age at blood draw.

	Age<50^a^			Age≥50^a^		
	n	Mean±SE (z score)	P for trend	n	Mean±SE (z score)	P for trend
**Nightshift (years)**						
Never	170	0.19±0.08	0.25	1413	−0.03±0.03	0.55
1–2	126	0.24±0.10		839	0.01±0.03	
3–5	58	0.30±0.14		611	−0.03±0.04	
6–9	23	−0.10±0.23		281	0.04±0.06	
10–19	29	0.10±0.21		276	−0.08±0.06	
≥20	6	−0.29±0.45		160	−0.05±0.08	
P for interaction, 0.29						
**Sleep duration (hours)**						
≤6	86	−0.03±0.12	0.004	940	−0.05±0.03	0.33
7	208	0.18±0.08		1495	−0.01±0.03	
8	80	0.31±0.12^b^		868	−0.008±0.03	
9	19	0.72±0.25^c^		148	−0.009±0.08	
P for interaction, 0.005						

a Adjusted for age (continuous variable), BMI (continuous variable) and cigarette smoking (pack years).

b The difference between ≤6 h and 8 h was statistically significant (P = 0.04).

c The difference between ≤6 h and 9 h was statistically significant (P = 0.007).

In addition, we found that women with the longest history of rotating night shifts work (more than 20 years) tended to have short telomere lengths compared with those with the shortest history of rotating night shifts work. This trend, while overall not statistically significant (P for trend  = 0.36), appeared to be more pronounced when restricting to women less than 50 years old, but the test for interaction between rotating night shifts and age was also not statistically significant (P for interaction  = 0.29). Overall, there was no association between night shifts and telomere length in this study. We examined the interactions between sleep duration and night shifts and BMI and smoking on telomere lengths, and none of the interactions were statistically significant.

## Discussion

Telomere length has been identified as a potential biomarker of aging [1]. Our previous study examined the effect of associations between diet, body composition, and lifestyle factors on telomere length in the NHS [22]. In the present study, we examine the associations of both rotating night shifts and sleep duration with telomere length. Our results suggest that sleep duration is positively associated with telomere length, except for those with more than 10-hour sleep. Individuals with long sleep duration (10+ hours) had shortened telomere length due to two possible reasons. Long sleep duration is likely to be an indicator of poor physical condition. Previous research showed that long sleep (≥9 hours) was associated with decreased physical performance in older women compared with mid-range sleepers (7–8 hours) [23]. On the other hand, people who sleep more than 10 hours can be very athletic. Our study showed that people with high level of moderate or vigorous physical activity had shorter telomere length than those with modest level (unpublished data).

Short sleep duration has been associated with a decreased risk of Parkinson's and increased risks of various cancers [12], [15], [24]. Three cohort studies have suggested that breast cancer risk decreased with increasing sleep duration. [12], [25], [26], but in our own cohort we did not observe an association between sleep duration and breast cancer risk [27]. Another study also reported an association between short sleep duration and an increased risk of colorectal adenomas [20]. Individuals sleeping fewer than 6 hours per night had an almost 50% increased risk of colorectal adenomas (OR = 1.47, P for trend = 0.02) compared with individuals sleeping at least 7 hours per night [20]. Similarly, with the exception of Parkinson's disease and melanoma, short telomere length in PBL has been suggested to be associated with an increased risk of breast, bladder, lung, and stomach cancers [4], [5], [28], [29]. Therefore, we hypothesized that there may be a positive association between sleep duration and telomere length. The current study supports the hypothesis that sleep duration is positively associated with telomere length, but the mechanisms underlying the association are still unknown. Melatonin, a hormone closely related to sleep, has been demonstrated to defend against oxidative stress, promote DNA repair and inhibit tumor development [30]–[32]. Some studies have shown that short telomere length is associated with high oxidative stress [33]. Recently, a link between short sleep duration and low levels of melatonin has been suggested [26], [34]. In a cohort study of sleep duration and breast cancer, the levels of melatonin in urine exhibited a dose-dependent positive association with the number of hours of sleep [26]. Another study also showed that serum melatonin levels were lower in individuals with<6 hours of sleep than those with >9 hours [34]. Therefore, sleep duration probably influences telomere length via a melatonin-mediated pathway. The finding that patients with obstructive sleep apnea syndrome had both an abnormal melatonin secretion pattern [35] and short telomere length [7] was compatible with this hypothesis. More studies are needed to elucidate the possible mechanism.

We observed that long sleep duration was significantly associated with long telomere length among women younger than 50 years old but not among those over 50 years old. Because telomere is shortened over age, it is possible that telomere attrition is more pronounced and more sensitive to environmental stress in early age and the attrition becomes slower over time. In addition, the effect of sleep duration on telomere length may be mediated through a melatonin-related pathway. High melatonin levels are a recognized protective factor against oxidative stress [30] and long sleep duration has been linked to high melatonin levels [26]. A significant trend of decreasing melatonin levels with increasing age was observed previously [36]. The variation of amplitude (night-day ratio of melatonin) was bigger in younger individuals than in older ones [36]. Our previous study also observed that younger women appeared to have higher urinary melatonin levels than older women, particularly in premenopausal women (≤39 yr versus ≥49 yr) [37]. Therefore, it would be predicted that the association between sleep duration and telomere length is more apparent among younger women.

Longer years of rotating night shifts work have also been associated with increased risk of various cancers, such as breast, colon, prostate, and endometrium [10], [38]–[40]. However, in our previous study, we also suggested that both long duration of rotating night shifts work and short telomere length are associated with a decreased risk of melanoma [13], [14] and Parkinson's disease [15], [16]. We found that women with a longer history of rotating night shifts work tended to have shorter telomere length, but the association was not statistically significant. Consistent with our findings, a cross-sectional analysis of the Sister Study showed no association between work at night (P = 0.83), rotating nightshift work (P = 0.44) and telomere length [6].

In conclusion, in this large prospective cohort study, we found that sleep duration was positively associated with telomere length, especially in women under 50 years old. Women with longer duration of rotating night shifts tended to have short telomere length, but it was not statistically significant. To our knowledge, this is the first study to report associations between rotating night shifts, sleep duration, and telomere length in a cohort study. Further research is needed to confirm these possible associations.
